# Exploring late Pleistocene bioturbation on Yermak Plateau to assess sea-ice conditions and primary productivity through the Ethological Ichno Quotient

**DOI:** 10.1038/s41598-023-44295-0

**Published:** 2023-10-13

**Authors:** Akanksha Singh, Matt O’Regan, Helen K. Coxall, Matthias Forwick, Ludvig Löwemark

**Affiliations:** 1 Department of Geosciences, National Taiwan University, 106, 13-318, Taipei, Taiwan; 2 Department of Geological Sciences, Stockholm University, 106 91 Stockholm, Sweden; 3 Department of Geosciences, UiT The Arctic University of Norway, 9037 Tromsø, Norway

**Keywords:** Palaeoceanography, Palaeoclimate, Climate-change ecology

## Abstract

Central Arctic, interglacial intervals have traditionally been associated with diverse and intense bioturbation, and abundant foraminifera, interpreted as indicating relatively low sea-ice concentrations and productive surface waters, while glacial intervals, typically barren, support the inverse. In this respect, the Yermak Plateau is anomalous. Biomarker studies suggest that glacial intervals were characterized by comparatively open water, while interglacials are marked by severe sea-ice conditions. Here we study downcore Ethological Ichno Quotient (EIQ) variations in trace fossils and bioturbation to test the hypothesis that different ethological classes vary in accordance with late Pleistocene changes in sea-ice extent, with deposit feeders increasing during reduced sea-ice cover and chemosymbiotic traces increasing during periods of thick perennial sea-ice conditions. Our results generally demonstrate that the abundance of traces like *Planolites*, *Scolicia*, and burrows produced by deposit feeders increase during episodes of seasonal sea-ice cover. In contrast, intervals with more severe sea-ice conditions are characterized by chemosymbiotic traces such as *Chondrites* and *Trichichnus*/*Mycellia*, suggesting lower food delivery and poorly ventilated bottom water conditions. The study thus confirms previous reconstructions of sea-ice conditions on the Yermak Plateau during interglacials, demonstrating that bioturbation variation provides insights into bentho-pelagic coupling under variable sea ice regimes in the Arctic Ocean.

## Introduction

A prominent consequence of Earth’s recent warming is the melting of Arctic sea ice^1–5^. Since 1978, the areal extent of the arctic summer sea-ice cover has decreased by around 3% per decade, according to data from satellite remote sensing^6–8^. Although, during summer the sea ice extent has declined more dramatically than winter sea ice extent. Particularly, the Barents Sea, has exhibited notable reductions^9^. Sea ice plays an important role in the Earth’s energy budget, where melting of Arctic sea ice lowers the Earth’s albedo resulting in a positive feedback for global warming^10–13^. Despite its central role in the climate system and its alarming disappearance, we have only a fragmentary view of how Arctic sea ice evolved and changed over geological timescales using proxies like biomarkers, foraminifera and dinocyst assemblages, as well as sedimentary ancient DNA^14–17^. This is in part due to the limited set of proxy tools that are available to reconstruct sea ice from marine sediment cores and challenges with their interpretation.

Over the past 10–15 years tremendous progress has been made in reconstructing sea-ice extent using biogeochemical proxies such as organic biomarkers^18–21^. The organic biomarker most commonly used to reconstruct sea-ice variability is IP_25_. This lipid is derived from sea-ice diatoms that occupy the interstitial channels at the base of the sea ice^22–24^ and tend to be more prolific (higher IP_25_ concentrations) in situations of seasonal sea-ice cover. Consequently, enhanced values of IP_25_ are interpreted as conditions proximal to the sea-ice margin. In contrast, production of IP_25_ in open water or under perennial sea-ice is very low or absent, and therefore lower IP_25_ concentration depicts such conditions, with the caveat that zero IP_25_ signal could equally be explained by thick perennial sea ice cover^22,25,26^. The use of these organic biomarkers, especially IP_25_, has become common recently^27–29^ compared to traditional proxies for sea ice, such as planktonic and benthic microfossil abundance^30,31^, and studies on ice rafted debris^26,32^. Foraminiferal assemblages and abundance are also useful proxies for reconstructing sea-ice variability, as they are generally abundant under open-water conditions, but scarce during periods of perennial sea-ice cover^33,34^. However, compared to other ocean basins, nanno- and microfossils, including calcareous and siliceous forms, are generally scarce in Arctic Ocean sediment records and show large spatial and downcore differences in preservation^35^. There are longstanding debates on whether this absence is a primary depositional signal (harsh environmental conditions), or results from dissolution at or below the seafloor as a function of organic matter supply and subsequent remineralization^36–38^. For example, in the central Arctic Ocean, there is seemingly better preservation of calcareous microfossils below perennial sea-ice cover where organic supply and microbial sedimentary CO_2_ is lower compared to the more productive ice-margin environments^39^, suggesting that there is a significant preservational bias.

The central Arctic Ocean is typically conceived as being covered with thick sea ice during glacial periods, with more open water conditions during the interglacial periods^14,34,40^, presumably allowing for increased surface primary production, organic carbon export to the seafloor and thus bioturbation in the sediment column. However, on the Yermak Plateau, which is located next to Fram Strait, the major gateway for Atlantic waters entering the Arctic Ocean, the opposite seems to be true. Glacial periods, which are well-dated in marine sediment cores from this region, are characterized by seemingly open waters while interglacials are characterized by perennial sea-ice conditions^21,41,42^. The presence of seasonal sea-ice conditions on the Yermak Plateau and the Barents-Kara margin during glacials is argued to arise from the formation of a coastal polynya system in front of the Barents ice sheet^21,41–43^. Here we investigate downcore variations of bioturbation in combination with trace fossil diversity on the Yermak Plateau. Importantly, a basic assumption is that the diversity of bioturbation and trace fossil types is directly influenced by the flux of particulate food falling from surface waters^44,45^ (i.e. export production and bentho-pelagic coupling). Consequently, we typically would expect higher bioturbation intensity under less severe sea ice or more open water/ice marginal environments, where photosynthetic primary productivity is higher^44–46^. Since sea-ice conditions on the Yermak Plateau during the past two glacial cycles are relatively well documented and the age models on glacial-interglacial timescale are robust^21,47,48^, it provides an excellent opportunity to test the use of bioturbation and trace fossils as a sea-ice proxy.

The aim of our study is to explore the quantitative analysis of bioturbation and trace fossils in sediment cores as an additional proxy-approach for exploring Pleistocene sea-ice history in the Arctic Ocean. The occurrence and activity of benthic fauna responsible for producing traces in deep-sea sediments depends on food availability, sediment substrate, bottom-water currents and oxygenation^44^, which in the Arctic Ocean are all strongly influenced by sea-ice conditions. The findings from the trace fossil analysis are compared to published sea-ice biomarker proxy data. Bioturbation and trace fossils have the immediate advantage of being largely resistant to diagenetic processes and are typically preserved in-situ in undisturbed sediment cores.

**Figure 1:**
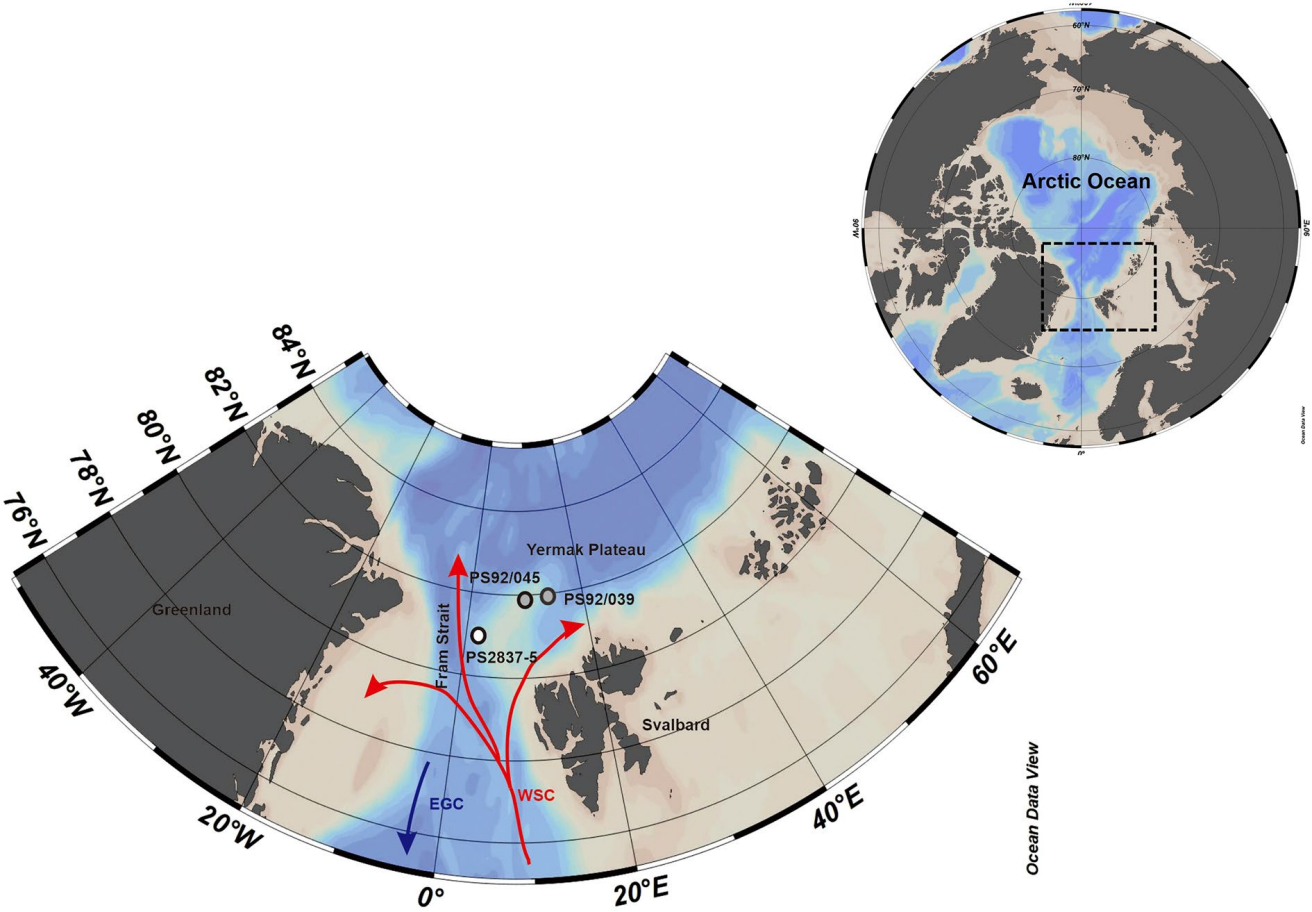
Location map showing the positions of the two studied cores PS92/039 and PS92/045 represented by grey dots, along with the position of core PS2837-5 from the western Yermak Plateau marked by a white dot. Red arrow shows the entrance of warm Atlantic water into the Arctic Ocean and indicates the West Spitsbergen Current (WSC). The blue arrow refers to the East Greenland Current (EGC). Cold polar water and sea ice departs the Arctic Ocean via southward streaming EGC. The map was created using Ocean Data View 2021 Reiner Schlitzer software (5.5.2) (https://odv.awi.de).

The studied cores are obtained from the Yermak Plateau (YP), a submarine high situated northwest of Svalbard (Fig. 1). The Yermak Plateau is adjacent to the Fram Strait, the only deep-water connection between the Arctic and Atlantic oceans, and is thus well positioned to monitor Arctic–Atlantic ocean exchange. Warm Atlantic Water (AW) flows northwards through this passage along the western coast of Svalbard and cold Arctic water flows southward along the eastern coast of Greenland^49^. This warm AW is the primary source of heat for the deeper Arctic Ocean basin^50^. After entering Fram Strait, the warm AW in the West Spitsbergen Current (WSC) branches out reaching the Yermak Plateau^51^, where the flow of AW is confined between 200–500 m depth^52^. At about 80°N WSC branches up into the Svalbard Branch and the Yermak Branch. The Svalbard Branch follows the continental shelf northeastward, and eventually sinks into the deeper Arctic^51,53^. The Yermak Branch flows northwest until about 81°N, and then east around the north western corner of the YP and eventually southward in the return Atlantic current^54^. The seafloor below about 700 m is bathed by Arctic Deep Water comprising some combination of northward flowing Nordic Seas Deep Water and ‘modified’ Nordic Seas Deep Water, which exits the Arctic after circling the Eurasian Basin^55^.

## Results

### Sediment type and color

**Figure 2:**
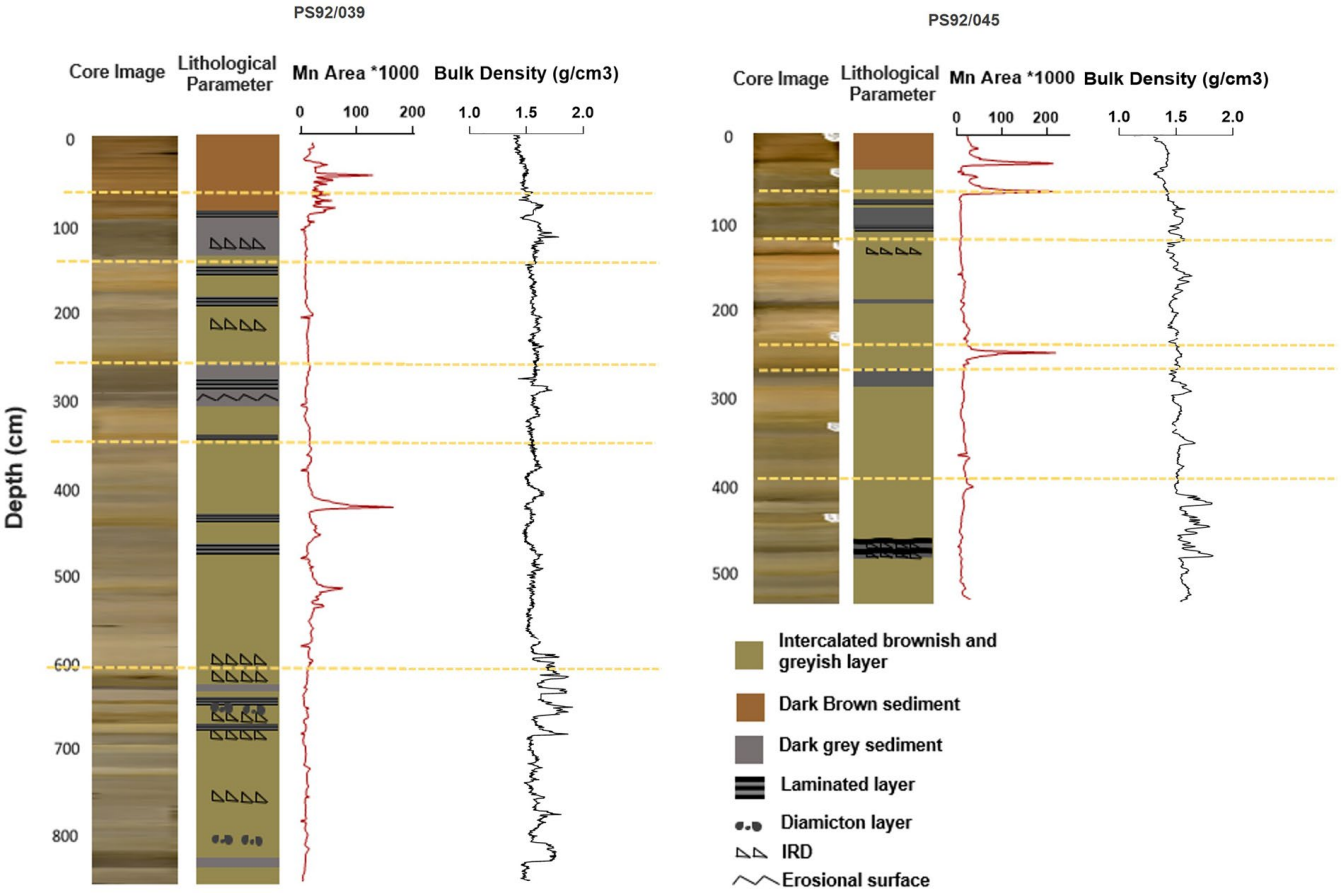
Variations in lithological parameters plotted along with Mn and bulk density (g/cm^3^) variations in studied cores PS92/039 and PS92/045 from Yermak Plateau. Yellow dotted line shows the division of different Marine isotope stages (MIS). Sedimentary structures (i.e., laminations, IRD etc.) shown are defined from the X ray images. IRD: ice rafted debris.

Both cores display alternating layers of brownish, dark brownish, greyish and dark greyish sediment with silty clay as the dominant lithology. The top layer of both cores is composed of a dark brown layer, corresponding to higher Mn concentrations. Several intervals with enriched Mn can be seen as distinct peaks in the XRF-scanning data (Fig. 2). However, the quasi-cyclic occurrence of Mn-rich layers, a common feature of the central Arctic Ocean sediments^56–59^, is not observed in the lower part of these cores (Fig. 2).

The visual core descriptions, environmental rock magnetic data and high resolution physical property measurements (bulk density) provide a definitive stratigraphic correlation between these two records (Fig. 2) and highlight a number of distinct lithological layers. Both cores contain three dark grey layers around 630–628 cm, 310–252 and 141–90 cm in core PS92/039, and 295–261 cm, 200–192 cm and 110–85 cm in core PS92/045 (Fig. 2). In PS92/039, laminated clay-silt layers occur at depths around 682, 658, 468, 430, 338, 288, 190, 160 and 80 cm, mostly corresponding to intervals with little or no bioturbation. The more pronounced of these layers roughly correspond to intervals of laminated clay-silt lithofacies in PS92/045 (445–462 cm, around 109 cm, and at 77 cm).

In PS92/039, IRD-rich layers are present at the interval of 762–757 cm, 670–662 cm, 620–590 cm, 217–212 cm, 132–124 cm (Fig. 2). The layers generally correspond to intervals of enhanced levels of IRD in core PS92/045 at the intervals 462–445 cm and 157–146 cm, and are reflected in both cores by high frequency changes in sediment bulk density (Fig. 2). There are also diamict layers, which can be identified by the presence of poorly sorted deposits and numerous clasts. At the water depths of the coring sites, these layers most likely reflect unsorted melt out from icebergs or sea ice^60,61^. At the water depths of the coring sites, these layers most likely reflect unsorted melt out from icebergs or sea ice^60,61^. Moreover, in PS92/039, at the depths of around 685, 647, 629, and 300 cm, erosional or diagenetic layers are identified (Fig. 2). These layers lack direct counterparts in PS92/045. Despite these minor differences, the two cores display a strong lithologic covariance.

### Ichnological analysis

**Figure 3:**
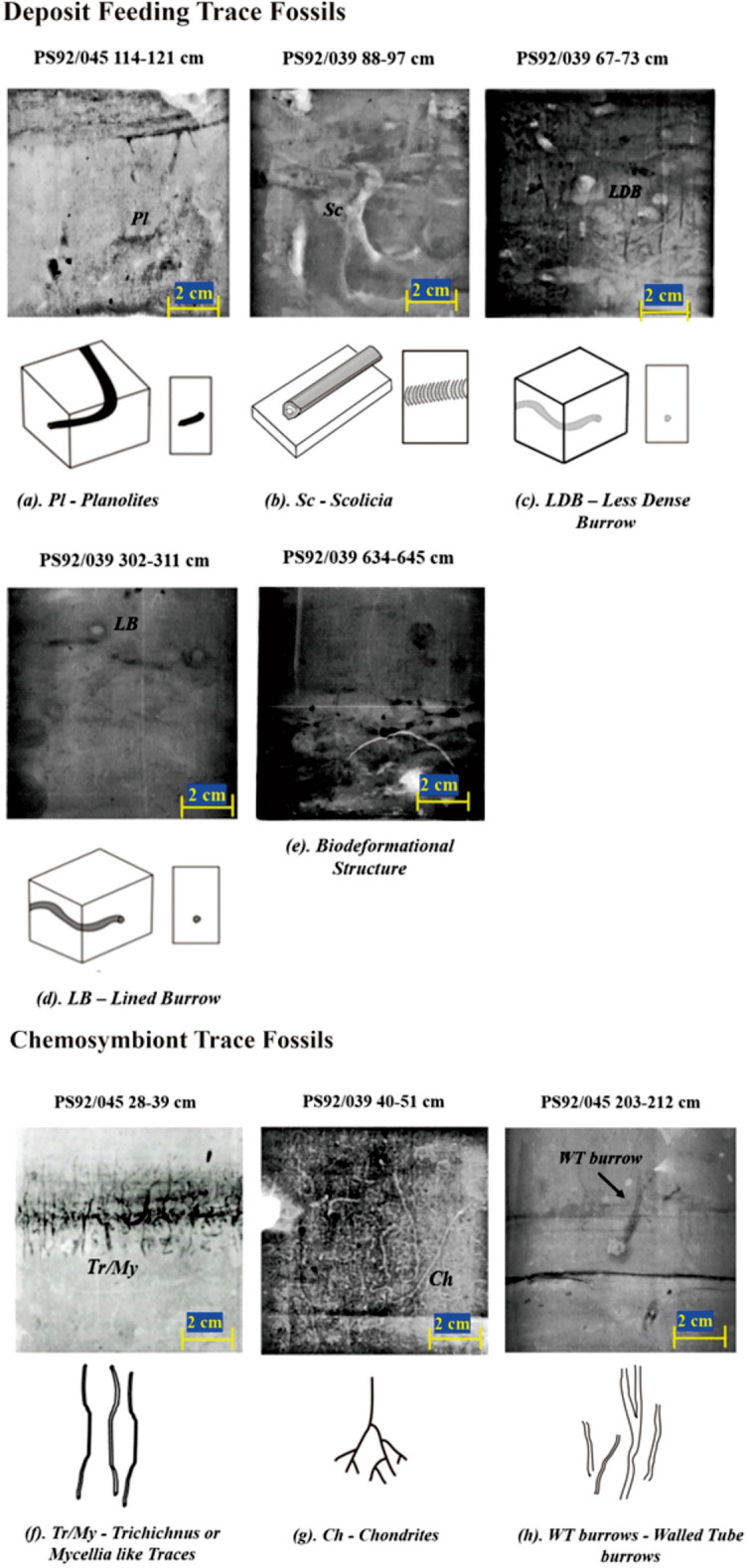
Common trace fossils observed in X-ray images of the two cores and illustrative cartoons that conceptualize the burrow morphologies. The trace fossils are classified under two main ethological classes, namely Deposit Feeding trace fossils: (**a**) *Planolites* (*Pl*); (**b**) *Scolicia* (*Sc*); (**c**) Less Dense Burrow (LDB); filling of the burrow is lighter that the surrounding material; (**d**) Lined Burrow (LB), has a distinct darker boundary; (**e**) Biodeformational Structure; and Chemosymbiont trace fossils: (**f**) *Trichichnus/Mycellia*-like Traces (*Tr/My*), dark filling indicates pyritized traces; (**g**) *Chondrites* (*Ch*); (**h**) Walled Tube Burrows. Tracefossil and burrow morphologies were traced out in CorelDRAW 2020.

Both cores show considerable downcore variability in the distribution and abundance of trace fossils. The major traces identified are *Planolites*-like, *Scolicia*-like, *Trichichnus*/*Mycellia*-like, *Chondrites*-like traces, Less Dense Burrow (LDB), where these LDB are characterized by less-dense material filling the interior while the outer boundary of the burrow is not well defined, and Lined Burrow (LB) (Fig. 3), as well as a few burrows that are not identified and are grouped under ‘Other Burrows’. Biodeformational structures, another common ichnological fabric, are seen in X-ray images of both cores. These represent regions that are completely mottled, although distinct traces cannot be recognized because of the high intensity of bioturbation and sediment mixing. Biodeformational structures are believed to reflect conditions of abundant food supply, where a specialization is not necessary^44^.

Overall, PS92/039 shows higher abundance of trace fossils along with a more diverse trace fossil fauna, containing ~ 8 different ichnotaxa, compared to core PS92/045, which contains ~ 6 different trace types (Supplementary Fig. 1).

**Figure 4:**
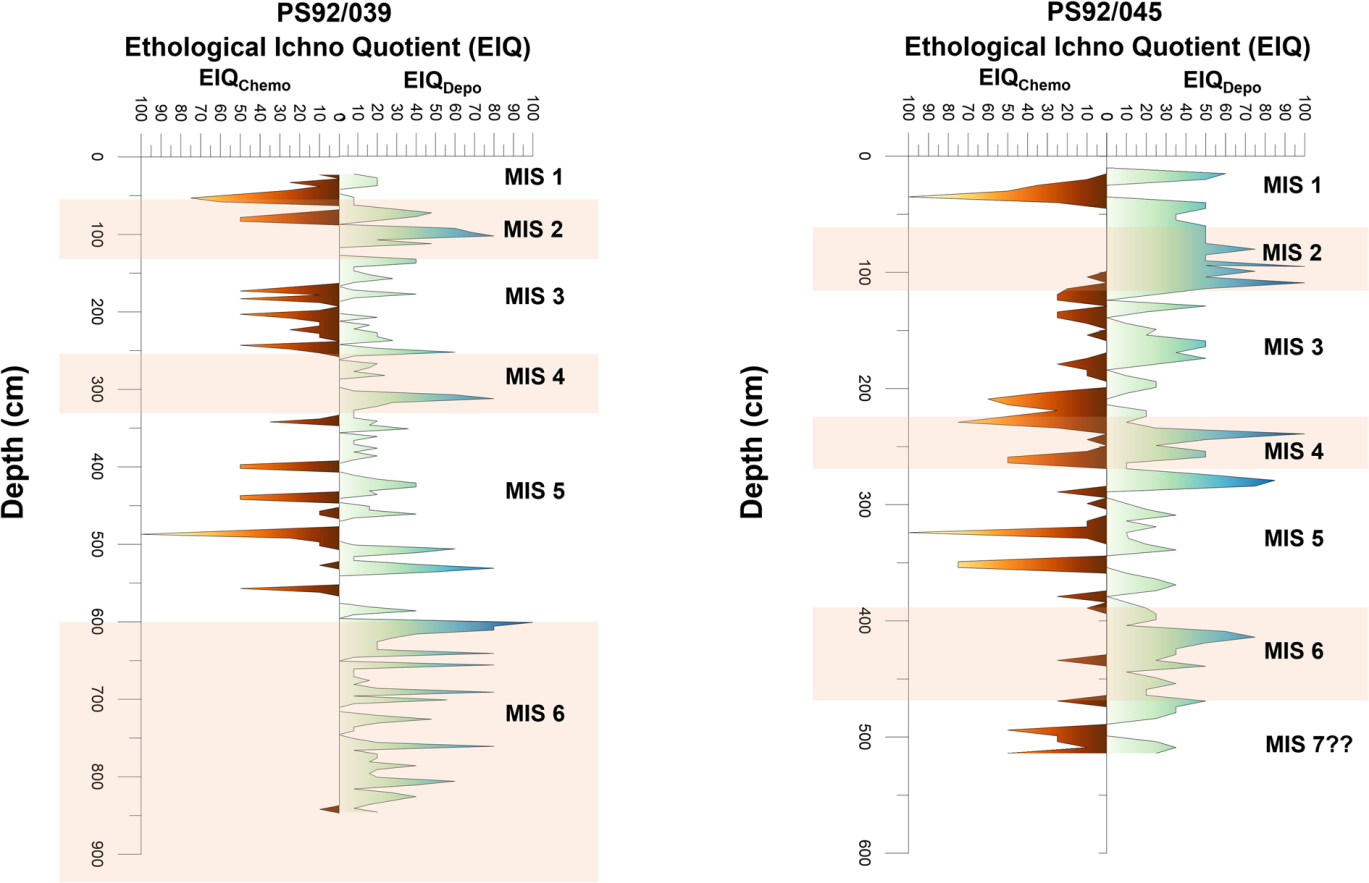
Ethological Ichno Quotient (EIQ) plot for deposit feeding and chemosymbiotic ethological-class trace fossils plotted versus depth for both examined cores PS92/039 (left) and PS92/045 (right). Orange shading indicates glacial periods.

*Planolites*-like traces are the most commonly observed trace fossils. *Trichichnus* and *Mycellia*-like trace fossils are also quite abundant in both cores, while rare *Chondrites* and *Scolicia* appeared only in core PS92/039 (Supplementary Fig. 1). To further dissect the relationship between the bioturbation and biogeochemical patterns, the trace fossils are divided into two main ethological classes, (i) chemosymbiotic traces and (ii) deposit feeding and dwelling traces (Fig. 3). Chemosymbiotic traces contains *Trichichnus/Mycellia*, *Chondrites*, and Walled Tube-like traces, in which the resident organism survives by forming a chemosymbiotic relationship with bacteria^62–64^. Deposit feeding trace, such as produced by *Planolites* or *Scolicia*, are produced by organisms extracting organic matter while ingesting the substrate^44,65^. Also, in some intervals mixed occurrences of chemosymbiotic and deposit feeder trace fossils are observed (Fig. 4).

For the two studied cores PS92/045 and PS92/039, a downcore Ethological Ichno Quotient (EIQ) index was generated for both deposit feeding and chemosymbiotic ethological class traces (Fig. 4). MIS 6 has rather intense bioturbation in both cores and is dominated by deposit feeding trace fossils, with an almost absence of chemosymbiotic traces in core PS92/039 and a few narrow peaks in core PS92/045 (Fig. 4). MIS 5 exhibits highly fluctuating conditions with the presence of both ethological classes in both the cores. The peaks of chemosymbiotic and deposit feeding traces primarily occur at alternating depths (Fig. 4). During MIS 4 core PS92/039 showed a dominance of deposit feeding traces, while core PS92/045 shows abundant traces of deposit feeding as well as chemosymbiotic traces mostly occurring at the same depths (Fig. 4). MIS 3 is dominated by chemosymbiotic traces in core PS92/039, with only two significant deposit feeding trace peaks occurring at the beginning and the end of MIS 3, while deposit feeding traces dominate in core PS92/045 in comparison to chemosymbiotic traces (Fig. 4). MIS 2 is dominated by deposit feeding traces in both cores, with a narrow interval of occurrence of chemosymbiotic traces in both cores. During MIS 1, significant occurrence of chemosymbiotic traces are found in both the cores. The EIQ plots of the two ethological classes indicate an abundance of deposit feeding traces during glacial periods (MIS 6, 4, and 2) and dominance of chemosymbiotic traces during interglacial periods (MIS 5, 3, and 1), as well as MIS 4 in core PS92/045. In general, the EIQ plots for the two cores correlate well with each other (Fig. 4).

## Discussion

**Figure 5:**
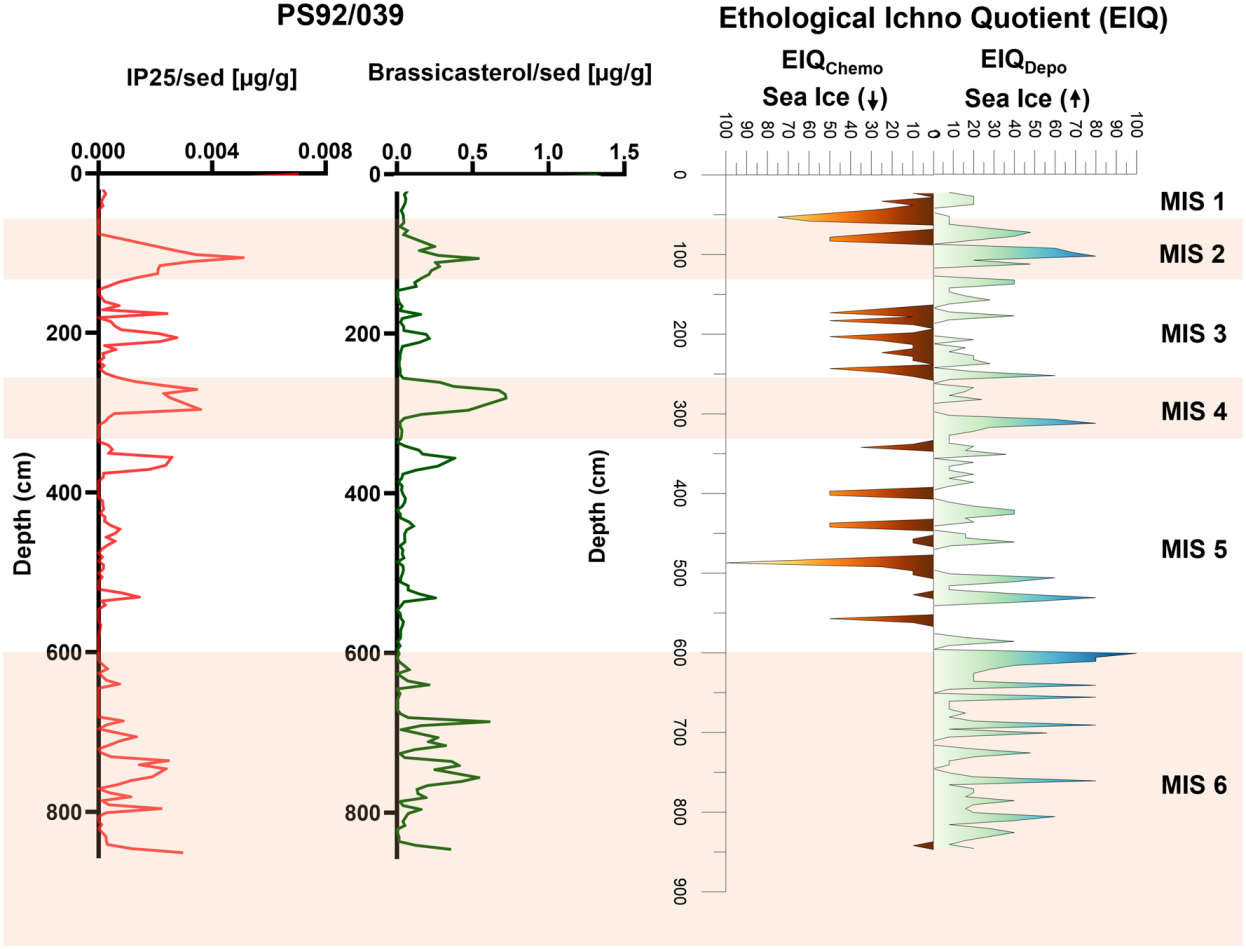
Comparison between the biomarkers: Brassicasterol (µg/g) and IP_25_ (µg/g) concentrations, from PS92/039 (replotted from Kremer et al.^21^, data obtained from PANGAEA—Data Publisher for Earth and Environmental Science, 10.1594/PANGAEA.884971) with Ethological Ichno Quotient (EIQ) plot from core PS92/039. Orange shading indicates glacial periods.

Generally, in the Arctic, we might expect low levels of bioturbation in glacial intervals due to low biological activity and productivity under thickened sea-ice cover. However, recent observations suggest that the Yermak Plateau was anomalous in this respect, with evidence for seasonal sea ice on its eastern flank even during glacial intervals, while interglacial periods are mostly characterized by severe sea-ice conditions^21,41,42^. This has been attributed to the combined roles of AW advection, which may have helped maintain seasonally open waters, while cold katabatic winds from the nearby Svalbard Ice Sheet fostered polynya development and sea-ice formation during glacials^21,41^. The downcore variations in the two ethological classes of trace fossils in both cores show a pattern with deposit feeding traces dominating during glacial periods in MIS 6, 4, and 2, indicating higher food-flux and well-oxygenated conditions, and chemosymbiotic traces dominating during interglacial periods MIS 5, 3, and 1, indicating poorly oxygenated, low food-supply conditions (Fig. 4). In general, the EIQ plot for both the cores exhibit a strong correlation with few notable differences in MIS 4 and MIS 2 (Fig. 4). This discrepancy might be attributed to the location of the two cores. Specifically, core PS92/039, with greater water depth is recovered from the location much closer to Svalbard. This proximity could render this core location more sensitive to ice sheet expansion and retreat than core PS92/045. The difference in water depth and proximity to Svalbard can potentially influence factors such as nutrient and oxygen availability, as well as biological activity which would affect the bioturbation intensity and diversity^66–68^. The variations in the occurrence and intensity of trace fossils from distinct ethological classes in core PS92/039 display strong similarities to patterns in the biomarker proxies (Brassicasterol and IP_25_) generated by Kremer et al.^21^ (Fig. 5). Variations in bioturbation and the abundance of the different ethological classes of trace fossils seem to support the conclusions drawn from previous studies on sea-ice indicators such as foraminifera, organic matter, biomarkers and mineralogical-based proxies. A detailed look at the individual glacial and interglacial stages reveals that trace fossils can offer valuable insight into pelagic-benthic coupling under past sea-ice conditions.

The Late Saalian (MIS 6) Ice Sheet was one of the Arctic's largest Quaternary glaciations^69^. According to previous reconstructions, the northern Svalbard Barents Ice Sheet (SBIS) should also have covered the Yermak Plateau during MIS 6^69–71^. Many studies of MIS 6 have indicated the recurrent waxing and waning of an unstable SBIS onto the outer shelf due to episodically intensified advection of warm AW^72–76^. The biomarker record from Kremer et al.^21^, showed higher IP_25_ flux and increased brassicasterol biomarker concentrations during most of the MIS 6 (Fig. 5) suggesting the presence of marginal sea ice cover at the Yermak Plateau at that time. Our bioturbation and trace fossil record document a similar pattern, where in core PS92/039 MIS 6 is largely dominated by deposit-feeder trace fossils throughout, indicating seasonal sea ice and well-oxygenated conditions (Fig. 5). This marginal sea-ice condition likely was driven by the formation of a coastal polynya along the northern Barents Sea margin, which may have been initiated by katabatic winds from the protruding SBIS and upwelling of relatively warm AW along the shelf break^32,77,78^. However, indications of erosion in MIS 6 in core PS92/39 (Fig. 2) could also raise the possibility of an incomplete record for this period. Past work has shown that the crest of the Yermak Plateau was eroded by glacial ice during MIS 6. Ploughmarks and glacial lineations are found at depth of up to 1000 mbsl^70,79,80^. No direct evidence exists for ice grounding at the coring stations, however grounded ice in proximal regions of the Yermak Plateau could have disrupted sedimentation.

Based on Kremer et al.^21^, biomarker and organic matter concentrations remain low throughout MIS 5 (Fig. 5). However, in contrast to the biomarker concentrations, throughout MIS 5 the trace fossils of core PS92/039 display alternating occurrence of chemosymbiotic and deposit feeding traces (Fig. 5). These variations indicate unstable sea ice conditions, with periods of severe sea ice conditions as well as periods of reduced or sea ice free conditions. This agrees with the IRD record along the Barents Sea margin^69,81,82^. Based on the IRD record, it appears that there were glacial advances during stadials of MIS 5, which were more limited than the MIS 6 glaciation. Five IRD peaks are recognized during MIS 5 in the region, and correspond to ice-sheet growth in the Barents and Kara seas during the MIS 5d and 5b transitions, as well as decay during the MIS 5d/5c and 5b/5a transitions^83^. The trace fossils of core PS92/039 suggest variable oceanographic or sea-ice conditions with occurrences of both chemosymbiotic and deposit feeding ethological classes alternating throughout MIS 5 (Fig. 5).

Previous research has shown that in comparison to MIS 6 and MIS 2, MIS 4 had a more restricted ice-sheet extent bordering the Yermak Plateau^77^. The biomarker record during MIS 4 displays an increase in IP_25_ and brassicasterol concentrations^21^. Our EIQ plot similarly demonstrates that deposit feeding traces predominate (Fig. 5), with the absence of chemosymbiotic traces. This is consistent with the idea of marginal sea-ice conditions caused by the formation of coastal polynyas in front of a MIS 4 ice sheet, as well as a northward movement of the sea-ice margin^41^.

MIS 3 was characterized primarily by high IP_25_ concentrations and significantly reduced brassicasterol values, indicating more severe ice conditions above the Yermak Plateau (Fig. 5)^21^. Furthermore, Svalbard continued to receive glacially eroded material along its western continental margin during most recent MIS 3, indicating that minor glaciations continued on Svalbard. However, looking at the EIQ plot, it shows that MIS 3 is the interval where chemosymbiotic and deposit feeding traces both show their occurrence simultaneously (Fig. 5). This co-occurrence of deposit feeding and chemosymbiotic traces could possibly be linked to localized sea-ice free conditions stemming from the continuous deglaciation of the Eurasian hinterlands during MIS 3^21^, which has been also shown by the presence of lower abundance of brassicasterol (Fig. 5).

The SBIS extended at the beginning of MIS 2. This was followed by a period of marginal sea ice, during which katabatic winds from the protruding ice sheet and/or enhanced AW incursion most likely resulted in the establishment of a coastal polynya north of Svalbard. The SBIS grew unstable after the Last Glacial Maximum, around 20 ka, when insolation was at its lowest^77,84^, supported by the presence of IRD peaks in the records from the Yermak Plateau at about 20 ka^32,73,77^. The detailed sea-ice variability study on core PS2837-5 (core location shown in Fig. 1) using biomarker proxies on the western Yermak Plateau by Müller et al.^85^ shows near absence of both IP_25_ and brassicasterol biomarkers between 30–20 ka, indicating the persistent existence of perennial sea ice cover over the western YP. This was followed by a gradual increase in both biomarker records, signifying a more favorable growth environment for ice diatoms and phytoplanktons and therefore, a transition to more seasonal sea ice conditions during late MIS 2. In the eastern YP core PS92/039, high brassicasterol and IP_25_ concentrations persisted for the majority of MIS 2 (Fig. 5)^21^, suggesting ice marginal conditions with seasonal sea ice and open waters. This is compatible with the dominance of deposit feeding trace intensity seen in the EIQ plot (Fig. 5). By the end of MIS 2, there appears to be a decrease in the abundance of IP_25_ and brassicasterol biomarkers and an increase in chemosymbiotic traces suggesting more severe sea-ice conditions as the SBIS retreated during deglaciation.

The establishment of perennial sea ice at the eastern Yermak Plateau appears to have occurred as a result of the final retreat of the ice sheet to the Svalbard coastline around 13 ka^82,86,87^, as illustrated by the declining biomarker contents in PS92/039 (Fig. 5)^21^. This decrease in biomarker concentration also coincided with the predominance of chemosymbiotic traces throughout MIS 1, indicating a state of low oxygen levels. This has been consistent with the few studies indicating that the occurrence of persistent sea ice cover during the late Holocene is a widespread phenomenon in Yermak Plateau^88,89^.

Overall, when viewed across the whole MIS stage, the variability in bioturbation observed on the Yermak Plateau through core PS92/039 shows a general consistent pattern with respect to glacial-interglacial changes in sea ice reconstructed from biomarker concentrations. The occurrence of the trace fossils formed by deposit feeders, such as *Planolites*, *Scolicia* and other distinct burrows generally correspond to the biomarker-based reconstruction of expanding marginal sea-ice conditions. However, deciphering the short-term variations in these ethological groups and establishing a direct peak-by-peak comparison between biomarker-based reconstruction and bioturbation study within a MIS stage could be challenging due to the limitation imposed by the available age model and the slight disparity between the two proxies. Bioturbation includes biogenic mixing or reworking of the sediment, involving process such as ingestion, locomotion, respiration and burrowing^90,91^. So, the trace fossils inherently have varying offset between the stratigraphic depth corresponding to their time of living, and the depth where they are preserved and therefore, bioturbation based reconstruction might represent the condition of the broader sediment depth interval, while biomarker and organic matter proxies provide information specific to particular depth. This study suggests that bioturbation and trace fossil analyses are of value in studying palaeo sea-ice conditions, adding an additional paleoceanographic tool for regions such as the Arctic Ocean, where micro and macro fossils are often scarce. Our bioturbation records point to alternating phases of enhanced and reduced food supply to the seafloor on the Yermak Plateau during MIS 5, suggesting large millennial-scale variability in seasonality impacting sea-ice conditions. Generally, the patterns of bioturbation suggest a greater variability in sea-ice conditions within individual Marine Isotope Stages than previously documented through biomarker studies.

Based on detailed studies of late Quaternary variations in trace fossils and organic biomarkers on the Yermak Plateau, the following conclusions can be made:

- Variations in the abundance of different ethological classes of trace fossils show pronounced changes across glacial-interglacial periods, with deposit feeders dominating during glacial intervals, and chemosymbiotic trace fossils dominating during interglacials.
- Variations in trace fossils can be effectively assessed and visualized using the novel Ethological Ichno Quotient.
- Variations in the ethological classes of the trace makers show a strong correspondence to variations in organic biomarker proxies such as IP_25_ and brassicasterol.
- Both organic biomarker and the trace fossil records support the notion of open water conditions and increased primary productivity and food supply to the benthic realm across the Yermak Plateau during glacial intervals.

Consequently, variations in the ethological classes of the trace makers assessed through the novel Ethologica Ichno Quotient can provide a powerful additional tool to address trends and shifts in sea-ice cover in regions where other paleoceanographic proxies are scarce or diagenetically overprinted. However, bioturbation abundance, diversity and distribution of different ethological classes within Arctic sediment along with it’s correlation to sea ice conditions, are influenced by a range of biological and environmental factors. Further exploration and discussion of these factors are essential to comprehensively understand the complete spectrum of variations in bioturbation intensity and diversity. The close correspondence between the abundance and diversity of bioturbation and biomarker-based reconstructions of productivity and sea ice on the Yermak Plateau warrant further comparisons in other glaciomarine settings, like active marine terminating glaciers, and under perennial and seasonal areas of sea ice in the central Arctic.

## Materials and methods

### Core locations

The cores in this study were recovered during the *Polarstern* TRANSSIZ expedition using a gravity (PS92/045) and Kasten Corer (PS92/039)^92^ (Table 1). PS92/039 was retrieved from eastern flank of the Yermak plateau at a water depth of 1464 m and PS92/045 from the central region of the Yermak Plateau at a water depth of 915 m. This places both cores below the base of the modern Atlantic water layer (~ 600 m) in what is defined as Upper Polar Deep Water^47^. These cores are situated approximately 64 km apart from each other (Fig. 1; Table 1). Although originating from the same geographical location, the cores exhibit distinct characteristic due to their differing position. core PS92/039’s proximity to Svalbard and its greater water depth, in comparison to core PS92/045, result in the representation of two distinct local environment.

**Table 1:** Details of the studied core stations.

Core ID	Latitude (°N)	Longitude (°E)	Water depth (m)	Core recovery (cm)
PS92/045	81.89	9.76	915	520
PS92/039	81.95	13.83	1464	860

### Sedimentology and core scanning

Both cores were logged shipboard on a Multi-Sensor core logger to acquire high-resolution downcore profiles of bulk density and magnetic susceptibility. They were later scanned using the Aavatech XRF Core Scanner at the Department of Geosciences. Basic sediment parameters were assessed onboard^92^, and the lithology of the two cores was described by examining core images as well as X-ray images (see method below).

### Age model

**Figure 6:**
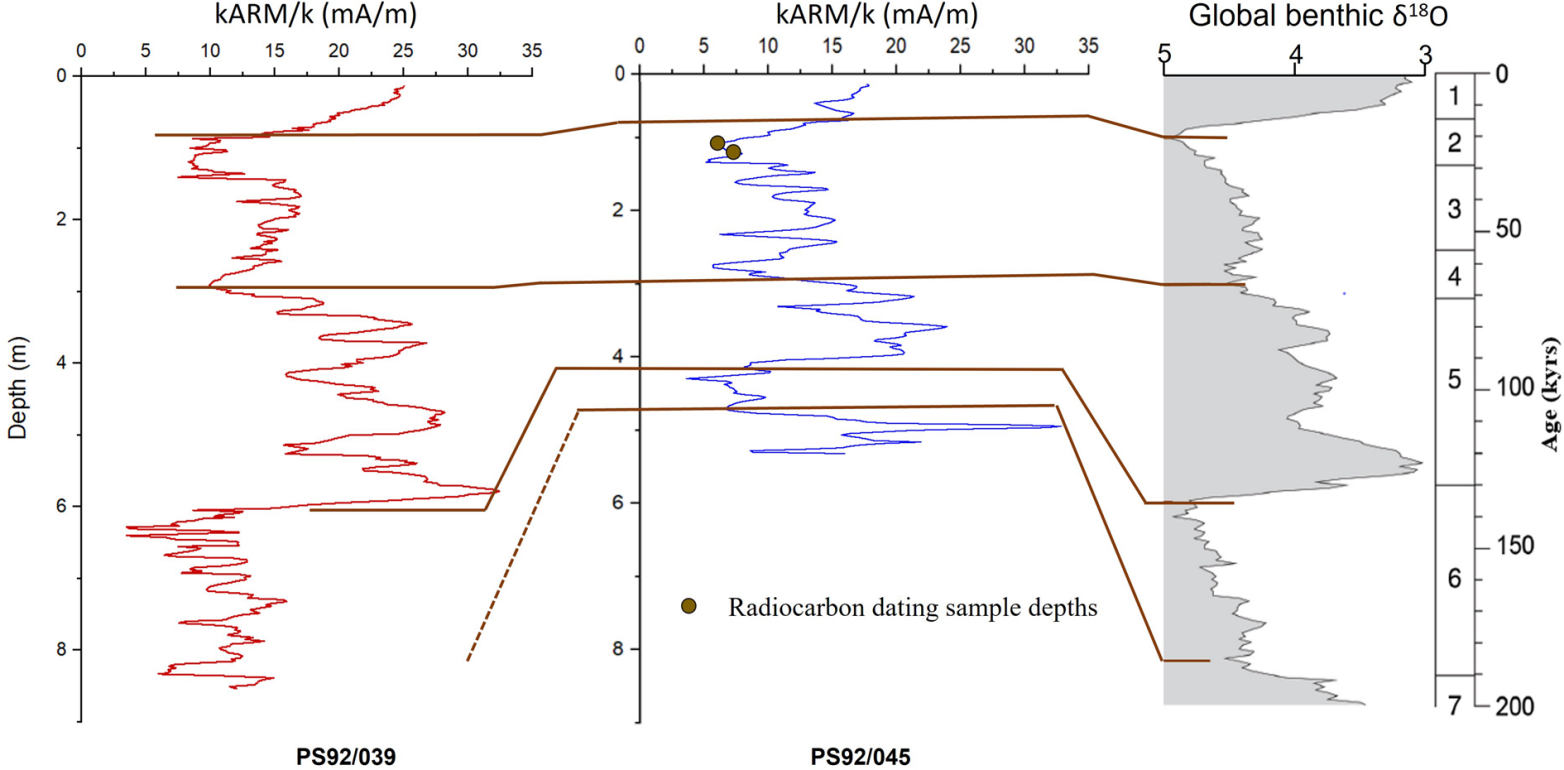
Lithostratigraphic correlation of core PS92/039 (left) and core PS92/045 (right) which forms the basis of the age model used here^47,48^. Correlation between environmental magnetic parameter k_ARM_/k (ratio between the anhysteretic magnetic susceptibility and the bulk magnetic susceptibility) variations of the two Yermak Plateau cores and the global benthic δ^18^O curve of Lisiecki and Raymo^94^ is shown by brown lines. Brown dots shows the two depths at which radiocarbon dates were measured in core PS92/045.

The age models for PS92/039 and PS92/045 were adopted from West et al. and Wiers et al.^47,48^. The age models primarily rely on a strong correlation between the rock magnetic parameters (i.e. the anhysteretic remanent magnetization normalized by the magnetic susceptibility) and the global benthic δ^18^O curve^47,48^. This correlation is supported by AMS^14^C dating, the occurrence of the benthic foraminifera *Pullenia bulloides,* used as a stratigraphic marker for Marine Isotope Stage (MIS) 5.1 (~ 81 ka) in the polar North Atlantic^93^, δ^18^O of planktonic foraminifera (PS92/045)^48^ and rates of amino-acid racemization in planktonic and benthic foraminifera^47^ (Fig. 6). Here we have added two new AMS^14^C dates for PS92/045 (Table 2) based on planktonic foraminifera. Two samples were prepared by separating 1 cm thick slices of sediment from core depths 115 cm and 128 cm, respectively. The sediment samples were sieved using a 63 µm mesh to separate the sand-sized sediment fraction containing foraminifera from finer grained material. Around 10,000 individuals of the planktonic foraminifer *Neogloboquadrina pachyderma*, weighing ~ 8 mg, were picked using the light microscope, cleaned, and prepared for radiocarbon dating at Accelerator Mass Spectroscopy Lab at National Taiwan University (Table 2).

**Table 2:** Radiocarbon ages of core PS-92/045. The dates were obtained on the planktonic foraminifera *Neogloboquadrina pachyderma.* Depth at which *N. pachyderma* foraminifera were collected are documented by using the unit cmbsf (centimeters below seafloor). Radiocarbon dates were adjusted to account for a 400-year reservoir effect and calibrated using CalPal Online Radiocarbon Calibration of 14C (http://www.calpal-online.de/)^95,96^. The calibrated ages have 1σ uncertainty.

Lab ID	Material	Depth (cmbsf)	Number of picked individuals	Weight (mg)	^14^C Age (year BP)	Calibrated Ages (year BP)
NTUAMS-5044	*N. pachyderma*	115	1150	8.4	16,218 ± 319	19,860 ± 450
NTUAMS-5045	*N. pachyderma*	129	1100	7.2	17,055 ± 351	20,820 ± 550

### Bioturbation and lithology analyses

The low contrast between the silty clays and the trace fossils made it difficult to discern variations in bioturbation and individual trace fossils from the split core surfaces using optical inspection/photography alone. Therefore, to evaluate variations in bioturbation and trace fossil content, X-ray radiographs were taken following the method described by Bouma^97^. To do this, sediment sub-cores were taken from the 30 × 30 cm and up to 6 m long gravity core, using a 25 cm long and 6 mm thick plastic box pressed into the split core to produce ‘X-Ray sediment slabs’. The sediment slabs were X-rayed at the X-Ray facility at the Alfred Wegener Institute for Polar and Marine Research, Germany. Variations in sediment density associated with the trace fossils relative to surrounding substrate disclose structures in the sediment that are otherwise invisible^98,99^. The radiographs were visually inspected and trace fossil morphologies traced out in CorelDRAW 2020 to identify and quantify all trace fossils. Sedimentary features like IRD-rich horizons, intervals with clear laminations and various other physical parameters revealed in the radiographs were also recorded.

The variations in recurrent biogenic sedimentary structures were studied in X-ray images from the two cores. Eight different kinds of recurrent biogenic structures were recognized. Taxonomically, it was generally not possible to unequivocally assign specific ichnospecies names to the observed trace fossils. Therefore, rather than assigning ichnospecies or ichnogenus names, we use generic terms, for example *Planolites*-like or *Chondrites*-like trace fossils (Fig. 4). The trace fossils and the bioturbation of the sediment were grouped into three main ethological classes representing different behaviors or environmental conditions (Table 3). The first ethological class consists of trace fossils representing deposit-feeding and dwelling behaviors and indicate well-oxygenated and food-rich conditions. The second ethological class includes chemosymbiotic trace fossils indicating dysoxic bottom waters and possibly low food supply. The third ethological class contains no biogenic structures but is dominated by primary sedimentary structures. This indicates very low food flux, or dysoxic conditions, inhibiting higher organisms from colonizing the substrate.

**Table 3:** The main ichnofabric types observed in cores PS92/039 and PS92/045, here defined as Ethological class 1–3. Behavioral associations for the different ichnotaxa follow Blanpied and Bellaiche; Fu and Werner; Wetzel^44,62,63,65^.

Ethological Class 1 Deposit feeders	Ethological Class 2 Chemosymbionts	Ethological Class 3 Laminated No bioturbation
*Planolites*-like *Scolicia*-like Less dense burrow Lined burrows Other burrows Biodeformational structures	*Trichichnus*-like *Mycellia*-like *Chondrites*-like Walled-tube burrow	Primary layering preserved, no ichnofossil observed

Variations in the abundance of the eight kinds of trace fossils were recorded at 5-cm intervals on an intensity scale ranging from 0 to 100, where 0 = absences of trace fossils; 10 = few; 25 = some; 50 = abundant; 100 = dominating. Downcore variations in trace fossil abundance and bioturbation intensity for both the chemosymbiotic and deposit feeding ethological classes were documented using the Ethological Ichno Quotient (EIQ), where EIQ is defined as a the sum of identified bioturbation intensities in the respective ethological classes, thus quantifying the degree or intensity of bioturbation visible in the X-ray radiographs. EIQ calculation for both the ethological classes is shown in Eqs. (1) and (2).

1 $${\text{EIQ}}_{{{\text{chemo}}}} = \Sigma \left( {{\text{EIQ}}_{{{\text{Chondrites}}}} + {\text{ EIQ}}_{{{\text{Trichichnus}}}} + {\text{EIQ}}_{{{\text{Mycellia}}}} } \right)$$

2 $${\text{EIQ}}_{{{\text{depo}}}} = \Sigma \left( {{\text{EIQ}}_{{{\text{Planolites}}}} + {\text{ EIQ}}_{{{\text{Scolicia}}}} + {\text{ EIQ}}_{{{\text{Less}}\;{\text{Dense}}\;{\text{Burrow}}}} + {\text{ EIQ}}_{{{\text{Lined}}\;{\text{Burrow}}}} + {\text{ EIQ}}_{{{\text{Other}}\;{\text{Burrows}}}} } \right)$$

### Supplementary Information

Supplementary Figure 1.

Supplementary Table 1.

## Data Availability

All data generated in this study are included in supplementary information. Supplementary information includes 1. Supplementary table giving EIQ values for all the traces fossils in the studied cores and 2. Supplementary figure showing trace fossil heat map for the trace fossils plotted versus depth for both examined cores.
