# Supplementary material for: Exploring late Pleistocene bioturbation on Yermak Plateau to assess sea-ice conditions and primary productivity through the Ethological Ichno Quotient
Source: Sci Rep. 2023 Oct 13;13:17416. doi: 10.1038/s41598-023-44295-0 (PMC10575951; doi:10.1038/s41598-023-44295-0)
Supplement: Supplementary file 1 — Supplementary Figure 1. [file 41598_2023_44295_MOESM1_ESM.docx]

| 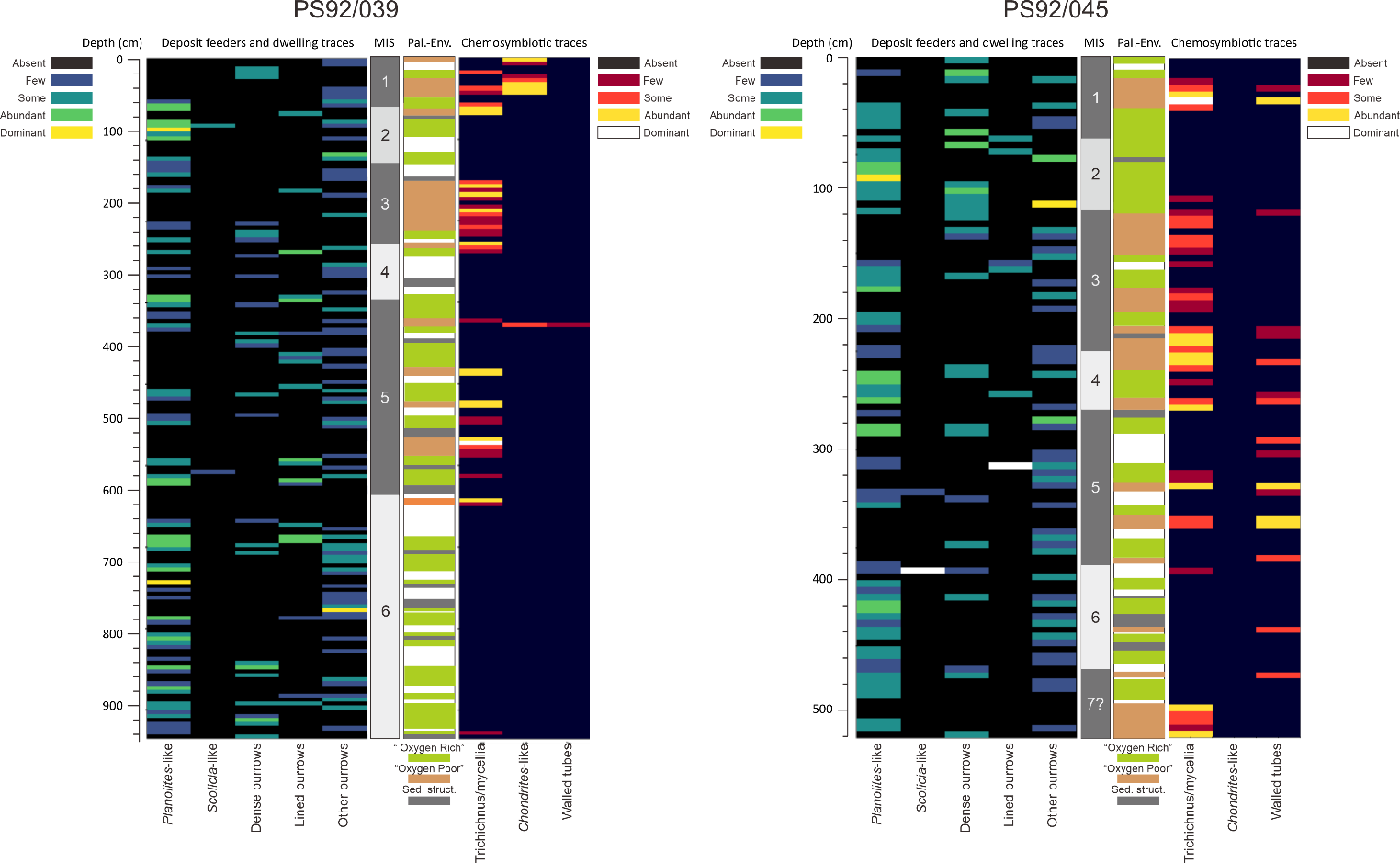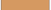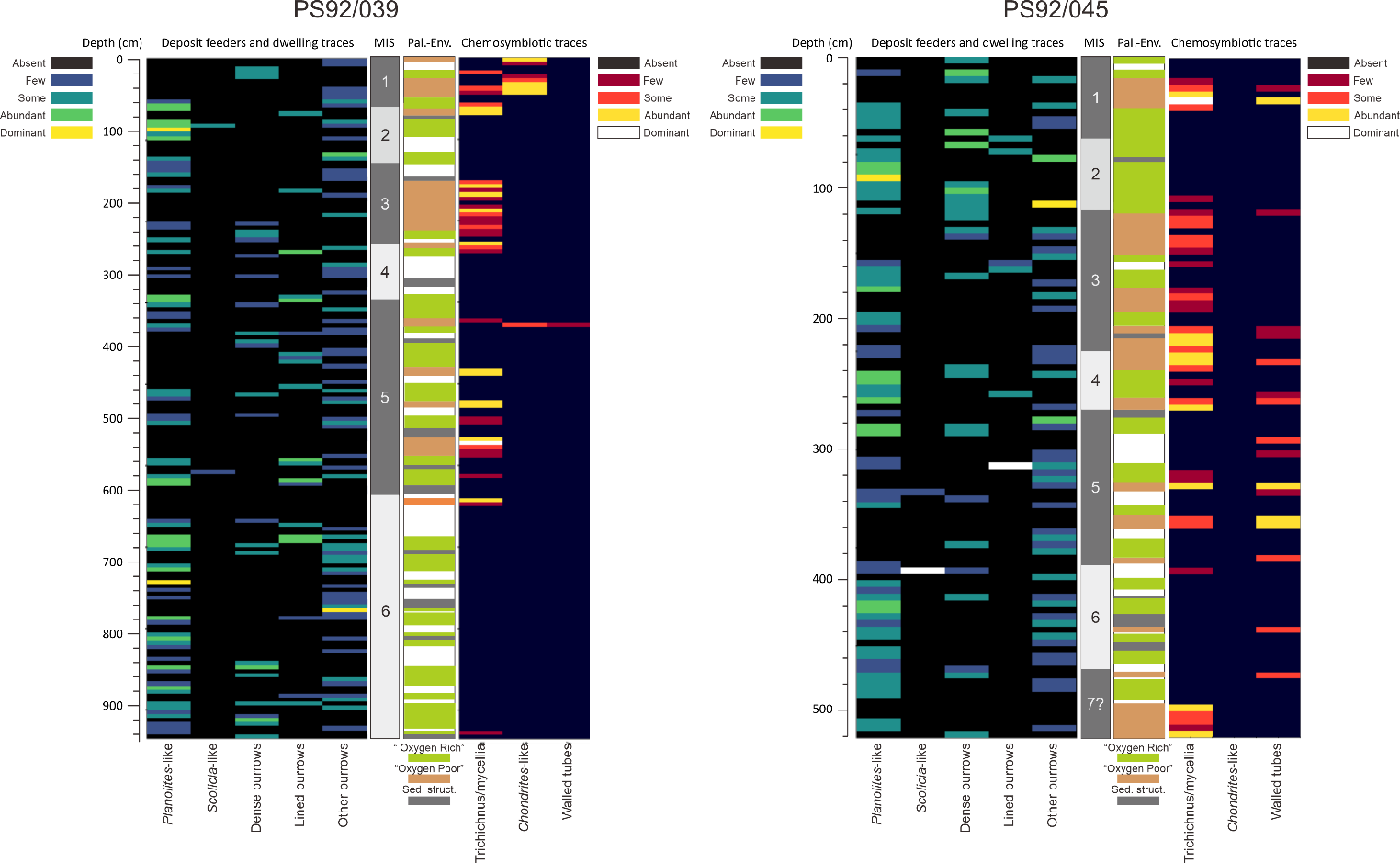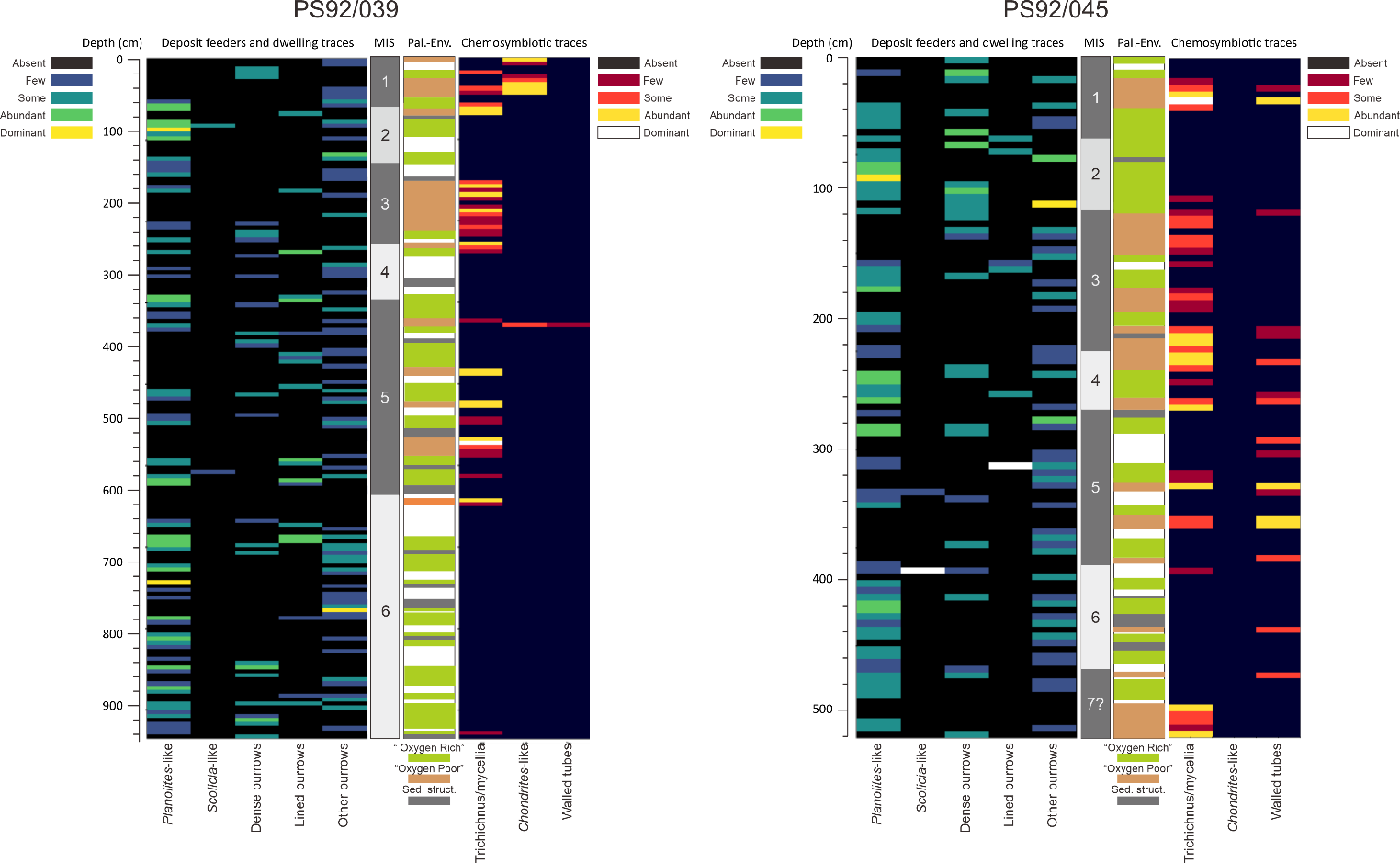 |  |  |  |  |  |  |  |  |  |  |
| --- | --- | --- | --- | --- | --- | --- | --- | --- | --- | --- |
|  |  |  |  |  |  |  |  |  |  |  |
| Supplementary figure 1. Trace fossil heat map for deposit feeding and chemosymbiotic ethological class trace fossils plotted versus depth for both examined cores PS92/039 (left) and PS92/045 (right). The heat map colors indicate the extent of bioturbation in individual traces. |  |  |  |  |  |  |  |  |  |  |
